# A Survey of Current Machine Learning Approaches to Student Free-Text Evaluation for Intelligent Tutoring

**DOI:** 10.1007/s40593-022-00323-0

**Published:** 2022-11-28

**Authors:** Xiaoyu Bai, Manfred Stede

**Affiliations:** Applied Computational Linguistics, University of Potsdam, Karl-Liebknecht-Straße 24-25, Potsdam, 14476 Germany

**Keywords:** Natural language processing, Deep learning, Automated essay scoring, Automated short-answer scoring, Intelligent tutoring systems

## Abstract

Recent years have seen increased interests in applying the latest technological innovations, including artificial intelligence (AI) and machine learning (ML), to the field of education. One of the main areas of interest to researchers is the use of ML to assist teachers in assessing students’ work on the one hand and to promote effective self-tutoring on the other hand. In this paper, we present a survey of the latest ML approaches to the automated evaluation of students’ natural language free-text, including both short answers to questions and full essays. Existing systematic literature reviews on the subject often emphasise an exhaustive and methodical study selection process and do not provide much detail on individual studies or a technical background to the task. In contrast, we present an accessible survey of the current state-of-the-art in student free-text evaluation and target a wider audience that is not necessarily familiar with the task or with ML-based text analysis in natural language processing (NLP). We motivate and contextualise the task from an application perspective, illustrate popular feature-based and neural model architectures and present a selection of the latest work in the area. We also remark on trends and challenges in the field.

## Introduction

Recent decades have seen increasing interest in modernising and digitalising education, not least due to the global Covid-19 pandemic, which has made traditional teaching methods impossible for lengthy periods of time in many parts of the world (Hesse et al., 2021; Gabriel et al., 2022). Stakeholders encourage the application of the latest technology to improving teaching and education.^1^

One of the main ways for artificial intelligence (AI) and natural language processing (NLP) to contribute is the development of automated assessment and tutoring tools that support teachers and students through evaluating students’ work and giving feedback on them. For instance, automated analyses of students’ essays can form the basis of formative feedback messages (Madnani et al., 2018; Zhang et al., 2019). Deployed in intelligent tutoring systems, such evaluation models can provide feedback in a timely manner even in a self-tutoring context where immediate feedback from teachers is not available. Studies on various use cases have emphasised the importance of such immediate feedback (Opitz et al., 2011; Marwan et al., 2020; Shute, 2008) as opposed to delayed feedback, which is given at a later point, such as days later.

While automatic assessment of multiple-choice tasks is easy, the automatic evaluation of free-form texts is a major challenge since the answer space is not pre-defined but unlimited in theory. However, tasks with free-form texts are highly desirable from an educational perspective: Instead of simply being able to recognise a correct answer, students are encouraged to learn by constructing correct answers for themselves and explaining their approach to problems (Rus et al., 2013). Moreover, writing longer essays not only allows students to develop essential writing abilities but also skills in critical thinking and judgement (Thu & Hieu, 2019; Fitzgerald, 1994).

In the present paper, we provide a survey of current machine learning (ML) and deep learning (DL) approaches to the automatic evaluation of students’ free-text production in the educational context, including short answers and essays. We refer to this task as *student free-text evaluation*. Depending on the task, students’ texts can range from short answers to question prompts that consist of few phrases (Maharjan & Rus, 2019) to short answers consisting of multiple sentences or a paragraph (Cahill et al., 2020), and finally to fully-fledged essays (Dong et al., 2017; Gong et al., 2021).

A few recent literature reviews have been published on related topics: Ke and Ng (2019) provide an overview on major milestones in the field of automated essay scoring. They discuss a set of selected works and present some of the frequently used datasets, but they do not cover recent approaches based on large language models like BERT. Beigman Klebanov and Madnani (2020)’s theme paper looks back at 50 years of essay scoring research and provides a high-level review of the research area without discussing technical details. Uto (2021)’s comprehensive review of essay scoring systems focuses on deep neural network approaches. It provides technical details on the architecture of a large body of neural systems but does not discuss their performance. An up-to-date systematic literature review on the field is given by Ramesh and Sanampudi (2021). They examine literature on essay scoring from the period 2010 - 2020, although works addressing short-answer scoring are also included. However, their review is limited to works on English data. With respect to short-answer texts, Galhardi and Brancher (2018)’s systematic literature review discusses over 40 papers on feature-based ML approaches to the task, i.e. approaches in which manually engineered features are fed to traditional ML models such as Support Vector Machines or Logistic Regression. However, their review is older and does not cover deep learning models. A more recent systematic review is provided by Blessing et al. (2021), which covers works from 2011 - 2019 but is also limited to feature-based ML models.

In short, our present survey differs from existing review papers as follows:

- We jointly discuss the evaluation of students’ essays and short answers since they can share a technical basis.
- Systematic literature reviews such as those mentioned above place emphasis on exhaustive search for literature and a methodical study selection process, while individual approaches are not explained in detail and little technical background is given. In contrast, we aim to provide an accessible survey of the field and do not assume familiarity with text evaluation tasks or deep knowledge of NLP other than general ML and DL techniques.
- We aim to present state-of-the-art approaches to the task. Therefore, we place particular focus on works published between 2019 and 2021, although some attention is also given to earlier, prominent work that often forms the basis of recent research. We include works on English and other languages.

Our survey is organised as follows: We first contextualise the task in intelligent tutoring applications in “Background: Student Free-Text Evaluation in Intelligent Tutoring Systems (ITS)”. In “Key Supervised ML Techniques in Student Free-Text Evaluation”, we describe prominent approaches to applying supervised ML and DL to the task and explain them with selected, concrete model architectures that illustrate the approaches. We consider traditional feature-based models (“Feature-Based Student Free-Text Evaluation”), neural architectures (“Neural Approaches to Student Free-Text Evaluation”) and hybrid models that combine both (1). We then present some of the latest work in the field with examples of use cases and data samples in “Recent Work on Student Free-Text Evaluation” and present state-of-the-art model performance on popular datasets in “State-of-the-Art on Popular Datasets”. “Conclusion” concludes our paper with a few general insights.

## Background: Student Free-Text Evaluation in Intelligent Tutoring Systems (ITS)

Intelligent tutoring systems (ITS) are a central application area for student free-text evaluation. This section looks at representative ITS which process, assess and respond to students’ natural language free-text production and for which accurate evaluation of students’ texts is the core prerequisite for tutoring. Example use cases cover both conversational and non-conversational ITS.

### Conversational ITS

Conversational ITS systems are based on tutorial dialogues in which the student interacts with a teacher-like virtual agent in natural language while discussing an educational topic or answering a set of questions (Olney et al., 2012; Rus et al., 2013; Graesser, 2016). To illustrate, the following example shows a chunk of tutorial conversation between a human student and a virtual agent tutor about a topic from physics, extracted and adapted from (Nye et al., 2014, p. 434):

**Tutor**: Suppose a boy is in a free-falling elevator and he holds his keys motionless in front of his him and he lets go. What will happen to the keys? Explain why. **Student**: The keys would not move but hover at the start point. **Tutor**: I bet you can explain this a little bit more. **Student**: The velocity of the keys is the same due to gravity. **Tutor**: What about the acceleration of the objects involved? **Student**: What is acceleration again?

Conversational ITS rely on a range of modules: Among others, the system needs to recognise the basic speech act of a student input, such as whether it is a request for help, a counter-question or an answer to the tutor’s question (Rus et al., 2013); when the student provides an answer in natural language, a natural language understanding (NLU) model needs to evaluate it based on pre-defined criteria (Graesser et al., 2004; Rus et al., 2013), which is where student free-text evaluation applies; and finally, a conversational system also needs a dialogue management model to track and navigate through conversation states (Graesser et al., 2004; Olney et al., 2012; Rus et al., 2013).

One of the most well known conversational ITS is AutoTutor (Graesser et al., 2004; Graesser, 2016; Nye et al., 2014), which has been applied to science and engineering subjects including conceptual physics and computer literacy. Based on curriculum scripts and anticipated correct and incorrect answers from students, AutoTutor provides educational dialogue by asking questions, evaluating students’ responses and subsequently giving students hints, motivational and formative feedback and explanations, among others (Graesser et al., 2004). Other prominent applications in a similar vein include DeepTutor (Rus et al., 2013), a conversational ITS for Newtonian physics, Guru (Olney et al., 2012), a system for high school biology, and ARIES (Cai et al., 2011), an ITS for training college students in scientific reasoning. A more recent example is Rimac (Katz et al., 2011; Albacete et al., 2019; Katz et al., 2021), another system for physics. Based on its assessment of students’ responses, Rimac provides feedback and models students’ individual knowledge levels in order to adapt to students’ individual needs.

### Non-Conversational Educational Applications

Alongside conversational ITS, a large amount of work centres on educational tools that automatically evaluate students’ work on different subjects and provide hints or feedback with respect to a specific task (Deeva et al., 2021; Nyland, 2018). In the absence of the dialog management task, the automatic evaluation of students’ work forms the central technological challenge for these tools.

Automated writing support (AWS) is one of the most actively researched areas related to educational tools: With respect to college-level students, Madnani et al. (2018) present Writing Mentor, a writing evaluation tool for scientific writing in English, which evaluates students’ texts along multiple criteria, including, among others, coherence, topic development, scientific conventions as well as orthographic and grammatical correctness.^2^ An extension of Writing Mentor to Spanish has recently been released (Cahill et al., 2021). Also working on Spanish, the system by González-López et al. (2020) specifically evaluates the methodology section of Mexican college students’ theses in engineering subjects. Argumentation skills in the writings of German-language business administration students are the target of the AL system (Wambsganss et al., 2020), which evaluates, among others, the coherence and persuasiveness of students’ argumentation and presents its findings in a dashboard view.

At the level of middle and high school education, eRevise (Zhang et al., 2019) gives formative feedback to English-language 5th and 6th-grade students on their short essays written in response to reading material. Another system targeting a similar age group is IFlyEA (Gong et al., 2021), which is a sophisticated essay assessment system for Chinese. Not only does it provide analyses on the levels of spelling, grammar and discourse structure, it also recognises figurative language and usage of various rhetorical devices and presents an overall feedback to the student in natural language.

Automated assessment systems are also of high significance to second language learning, where they are commonly referred to as intelligent computer-assisted language learning (ICALL): FeedBook (Rudzewitz et al., 2018; Ziai et al., 2018) is an ICALL system supporting middle school level English exercises. It recognises targeted grammar errors and retrieves tailored corrective feedback for each error type. TAGARELA (Amaral et al., 2011) is a comparable ICALL system for Portuguese. Other recent systems for learners of English include LinggleWrite (Tsai et al., 2020), which provides, among others, grammatical error corrections, writing suggestions and corrective feedback.

In the so-called STEM subjects (science, technology, engineering, mathematics), application examples include WriteEval (Leeman-Munk et al., 2014), which analyses and scores short-text responses by secondary students in science subjects. Kochmar et al. (2020)’s model, deployed in the learning platform Korbit^3^, evaluates data science students’ short-text answers to questions and provides personalised hints and explanations. Riordan et al. (2020) look into scoring secondary-school students’ textual responses to science questions according to the specific rubrics laid down by American educational authorities.

## Key Supervised ML Techniques in Student Free-Text Evaluation

The central component of both conversational ITS and other tutoring tools is the accurate and fine-grained evaluation of students’ natural language free-text production in response to a question, prompt or task formulation. In this section we zoom in on the key techniques used in supervised ML approaches to student free-text evaluation. We first look at ML approaches based on hand-crafted features and then turn to representation-based neural models as well as approaches using a combination of both.

In general, the set-up of using ML to assess students’ texts is straight-forward: An ML model takes the student text as input, possibly in combination with further textual information such as the task prompt or an expert reference answer. It then outputs a verdict about the input student text. A regression model is typically used when a score is the desired output verdict (Dong et al., 2017; Mathias and Bhattacharyya, 2020). Conversely, classification is used when the model output is a correctness judgement (Leeman-Munk et al., 2014), or when the model is designed to recognise specific writing components in the student text (González-López et al., 2020).

### Feature-Based Student Free-Text Evaluation

#### Feature Sets and Models

As is the standard approach in classical feature-based NLP, the main objective is to design an informative feature vector representation of the textual data sample and to feed the feature vector to a (supervised) ML model. The majority of effort thereby lies in selecting and engineering the most informative set of linguistically informed features, which depends on the concrete task to be learned. Thus, in student free-text evaluation, feature sets vary depending on the desired type of evaluation in a given use case. For instance, a system giving a holistic score for college-level social science essays will differ significantly from those used for recognising whether or not middle school students are providing the correct answer to a physics question.

For holistic essay scoring, some of the commonly used features are simple length-related features, such as the *essay length*, *average word length* or *average sentence length* (Nguyen & Litman, 2018; Phandi et al., 2015; Attali & Burstein, 2006). To capture lexical and sentence complexity, features include *number or percentage of stop words* (Nguyen & Litman, 2018), *word frequencies across words in the essay* (Attali & Burstein, 2006) and *text readability* features (Uto et al., 2020). In addition, the assessment of content and context in student texts frequently uses features such as *word n-grams* (i.e. chunks of *n* adjacent word tokens) (Riordan et al., 2020; Cahill et al., 2020) and *Part-of-speech (POS) n-grams* (Phandi et al., 2015; Kumar et al., 2020). Where assessment takes into account the task prompt to ensure that the student’s text is relevant to the prompt, *word overlap between the student text and the prompt* has been used as a feature set (Phandi et al., 2015; Nguyen & Litman, 2018; Kumar et al., 2020). Similarly, if reference answers or reference essays are available, *overlap or other comparative metrics between the student and the reference text* may constitute a key feature set (Meurers et al., 2011; Attali & Burstein, 2006; Leeman-Munk et al., 2014). Finally, for scoring texts by non-native speakers in particular, Vajjala (2018) provides detailed analyses of various linguistic features, including linguistic errors that are particularly significant for assessing learner texts.

As features are highly dependent on the concrete task and use case, the general features can be complemented by tailored feature sets that reflect systems’ evaluation goals in specific use cases. To illustrate, González-López et al. (2020) give feedback on methodology sections in college-level engineering theses, hence their feature set includes keywords that indicate the presence of a logical sequence of steps; Cahill et al. (2020) score student responses to mathematics questions where the responses contain mathematical expressions, and therefore they include the correctness of those mathematical expressions as a feature for the feature-based scorer. Moreover, Nguyen and Litman (2018) and Ghosh et al. (2016) find argumentation features to be useful for scoring *persuasive* student essays.

In terms of models, classical supervised classification and regression models are typically employed, including Support Vector Machines or Regressors (SVM / SVR) (Cahill et al., 2020; Johan Berggren et al., 2019; Horbach et al., 2017; Mizumoto et al., 2019), Linear Regression (Cahill et al., 2020), Logistic Regression (Nguyen and Litman, 2018; Johan Berggren et al., 2019; Ghosh et al., 2016), Random Forest classifiers (Mathias & Bhattacharyya, 2018; Kumar et al., 2020) and Bayesian Linear Ridge Regression (Phandi et al., 2015). Discriminative classification approaches seem to be favoured overall, although generative models like Naïve Bayes have also been used (Mayfield & Black, 2020).

#### Pros and Cons of Feature-Based Models

A major advantage of hand-crafted features is their human-interpretable nature such that analysing the features can yield interpretable insights: For instance, in their feature-based short-answer scoring task, Kumar et al. (2020) extract the importance of each individual linguistically-informed feature set and use it as a basis for feedback to the student. Moreover, feature-based approaches are useful when little annotated training data is available (González-López et al., 2020); Nadeem et al. (2019) additionally find that in a low-resource scenario, hand-crafted features can be particularly effective *in combination with* a neural architecture (see “Combination of Neural and Feature-based Models”). Finally, Ding et al. (2020)’s experiments on short-answer scoring in an *adversarial* setting suggest that classical, feature-based systems might be less susceptible to certain types of gaming and cheating attempts by students than end-to-end neural models are (see “Automatic Short-Answer Scoring (ASAS)”).

On the downside, models based on hand-crafted features require extensive feature engineering by domain experts, which is evidently costly. In addition, while some features, like average sentence length or the percentage of stop words, are easy to obtain, the automatic extraction of several features requires other existing NLP tools, e.g. POS-taggers for POS-tag features, syntactic parsers for syntax features or discourse parsers for discourse features etc.. Such tools must be available and adequately reliable for the language worked on. Moreover, complex features can be difficult to extract even for so-called *high-resource* languages, i.e. well researched languages in the NLP community for which data and tools are more easily available, such as English: For instance, extracting argumentation features relate to the field of argumentation mining, which is a challenge in its own right (Peldszus and Stede, 2016; Stab & Gurevych, 2017). Ghosh et al. (2016) find that while argumentation features are in principle useful for scoring students’ persuasive essays, the positive effect is compromised when argumentation features are extracted automatically due to errors at the argument mining stage.

### Neural Approaches to Student Free-Text Evaluation

In the past decade, end-to-end neural approaches have replaced feature-based ML and dominated most areas of NLP-related research, and the evaluation of student texts is no exception. Unlike in feature-based approaches, neural models *learn* a dense, non-interpretable vector representation of the input text(s) and feed it to an output classification or regression layer. Thus, the main challenge here is the design of a model architecture such that the most informative signals in the input text can be learned and encoded in a dense vector representation. We discuss and illustrate prominent neural architectures. While older, they often form the basis of recent work (“Recent Work on Student Free-Text Evaluation”).

#### Classical Neural Approaches: RNNs and CNNs

##### RNNs and LSTMs

Given the sequential nature of natural language, recurrent neural networks (RNNs)^4^ are an intuitive choice for encoding textual data and have been used in a large number of NLP models (Chen et al., 2017; Gong et al., 2019). More sophisticated RNN variants, such as the Long Short Term Memory (LSTM) (Hochreiter and Schmidhuber, 1997) and Gated Recurrent Units (GRU) (Cho et al., 2014), have been proposed to alleviate the issue of vanishing and exploding gradients during model training (Hochreiter, 1998). LSTMs, in particular, have become a standard choice for encoding language data, where model input is typically sequences of word or character tokens, and have achieved great results in NLP tasks like natural language inference (Nangia et al., 2017; Lan & Xu, 2018) and POS-tagging (Plank et al., 2016).

**Fig. 1:**
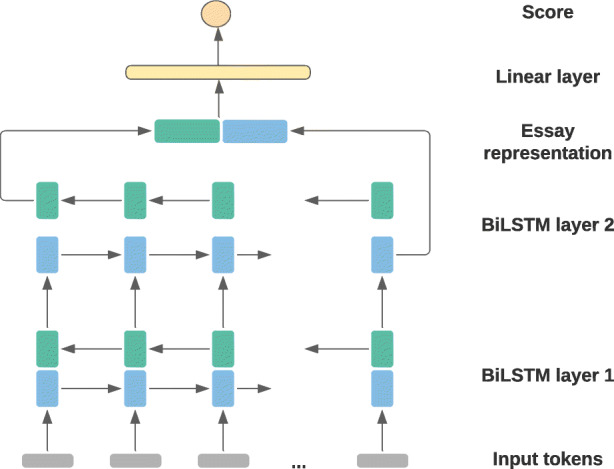
Two-layered BiLSTM essay scoring architecture adapted from Alikaniotis et al. (2016)

As is generally the case in RNNs, LSTMs can be extended to *bi-directional* LSTMs (BiLSTMs), which combines a forward and a backward LSTM and reads in the input sequence from both directions.^5^ Moreover, multiple layers of RNNs can be stacked on top of each other to form multi-layer RNNs for additional expressive power. In a multi-layer RNN, the hidden state vectors generated by a given RNN layer act as input vectors to the next RNN layer. A two-layered BiLSTM architecture is shown in Fig. 1.

Alikaniotis et al. (2016) was among the first to apply neural models to the automatic scoring of student essays. They experimented with a series of LSTM-based models and obtained particularly successful results with a two-layer BiLSTM architecture. In this approach, each student text sample was represented as a sequence of word tokens, represented by word vectors, that were fed to a two-layered BiLSTM encoder. Alikaniotis et al. (2016) concatenated the respective last hidden state of the forward and the backward LSTM of the second BiLSTM layer to obtain an encoding of the full text sample. This representation of the whole essay was then passed to a linear output layer for score prediction. Figure 1 illustrates their two-layered BiLSTM scoring model.

##### CNNs

Convolutional neural networks (CNNs) (Fukushima, 1979; LeCun, 1989) initially became particularly popular in tasks related to computer vision, such as handwriting recognition (LeCun et al., 1998; Wu et al., 2014) or image captioning (Xu et al., 2015). In recent years, they have also been shown to be successful in NLP tasks such as sentence classification (Kim, 2014; Gambäck and Sikdar, 2017).

**Fig. 2:**
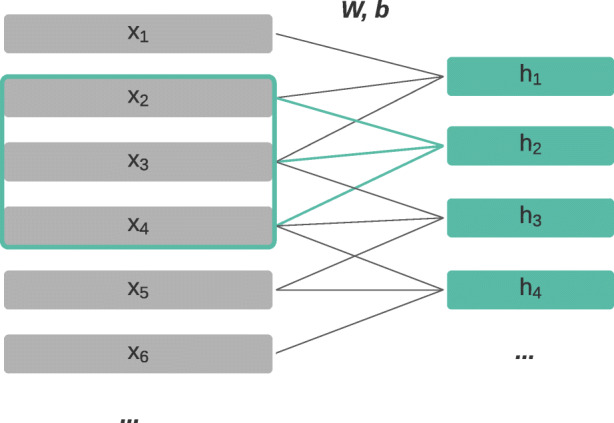
Convolutional extraction of trigram feature maps, where **W** and **b** are model parameters, indexed instances of **x** represent input tokens and indexed instances of **h** the extracted feature maps

CNNs employ a set of learned weight matrices of a pre-specified size (*convolutional filters*) and “slide” them step-by-step across the input data, applying a matrix multiplication at each step to extract local features (*feature maps*) from the input data.^6^ In the field of NLP (see Kim (2014) and Zhang and Teng (2021)), the input data consists of sequences of word (or character) token vectors. Moving along a sequence token-by-token, we take each context window of *k* tokens and apply matrix multiplication to the *k* token representations. The convolutional filter size is thus determined by the chosen context window size *k*. The filter moving over the input sequence then extracts a local feature map from each *k-sized n-gram*. As an example, the terms in (1), based on Zhang and Teng (2021), show the extraction of four trigram feature maps from the first six tokens of a sequence **x**_1_,**x**_2_,...,**x**_6_ with a window-size of *k* = 3,

1 $$ \begin{array}{@{}rcl@{}} \mathbf{h}_{1} &=& \mathbf{W}(\mathbf{x}_{1}\oplus\mathbf{x}_{2}\oplus\mathbf{x}_{3}) + \mathbf{b} \\ \mathbf{h}_{2} &=& \mathbf{W}(\mathbf{x}_{2}\oplus\mathbf{x}_{3}\oplus\mathbf{x}_{4}) + \mathbf{b} \\ \mathbf{h}_{3} &=& \mathbf{W}(\mathbf{x}_{3}\oplus\mathbf{x}_{4}\oplus\mathbf{x}_{5}) + \mathbf{b} \\ \mathbf{h}_{4} &=& \mathbf{W}(\mathbf{x}_{4}\oplus\mathbf{x}_{5}\oplus\mathbf{x}_{6}) + \mathbf{b} \\ ...&& \end{array} $$

where indexed instances of **x** denote input tokens, indexed instances of **h** denote each of the trigram feature maps extracted, **W** and **b** are the learned parameters of the convolutional filter, and ⊕ denotes concatenation. The feature maps extracted by convolution can be thought of as enhanced n-gram features which are learned and updated in the course of training. A visual representation of this same process is depicted in Fig. 2.

A pooling operation is typically performed to aggregate the extracted set of feature maps into a single vector representation **h**_*final*_ to encode the full input text. Common simple methods include maximum pooling and average pooling (Zhang & Teng, 2021). A more sophisticated alternative is pooling based on neural attention (Bahdanau et al., 2014). Without getting into details (see Zhang and Teng (2021) for a summary on attention pooling), the model learns individual attention scores for each feature map. Vector **h**_*final*_ is then computed by summing all feature maps, where each is weighted by its individual attention score. Attention pooling captures the intuition that some parts of the input text are more informative to the training task than others. In the case of student free-text evaluation, for instance, content words in a student answer are likely more informative for content-oriented evaluation than are function words such as articles and prepositions.

**Fig. 3:**
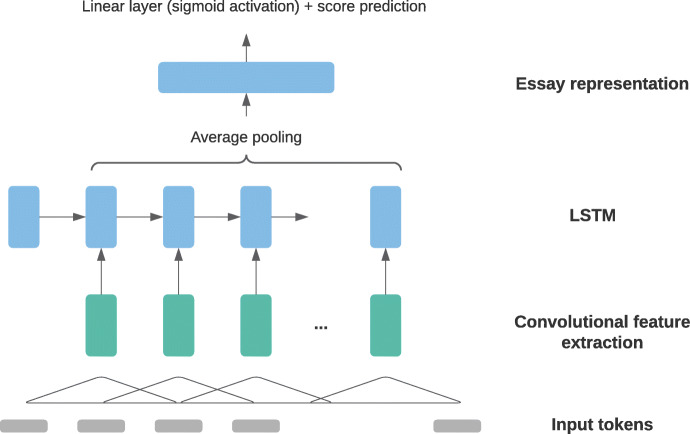
Convolutional-recurrent essay scoring architecture with average pooling, adapted from Taghipour and Ng (2016)

Taghipour and Ng (2016) conducted student essay scoring experiments with various architectures, including an influential architecture combining CNN and LSTM. In this model, a convolution layer first extracted local feature maps from the input word vector sequences based on a window size of *k* = 3; these feature maps were then fed to a single-layer LSTM. Thus, instead of directly taking word vectors as input, the LSTM took the output feature vectors of the convolution layer as input. Subsequently, Taghipour and Ng (2016) used average pooling across the hidden state outputs by the LSTM to obtain representations of full student essays, which were then sent to a linear layer with sigmoid activation for score prediction. Figure 3 illustrates this architecture.

**Fig. 4:**
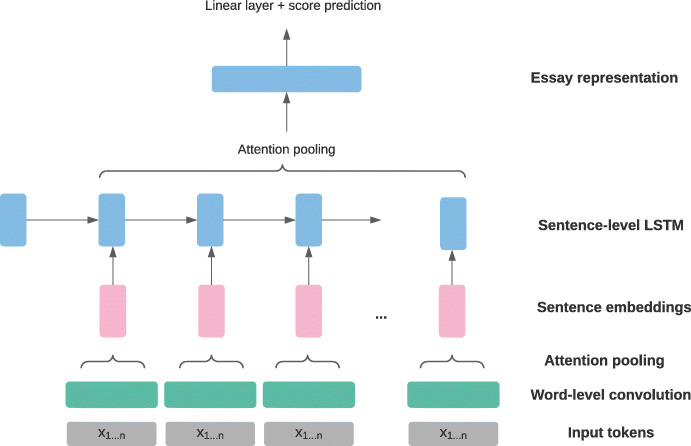
Hierarchical essay scoring architecture adapted from Dong et al. (2017) using explicit sentence-level representations and attention pooling; **x**_1...*n*_ denotes an input sentence consisting of *n* tokens

Another well-known convolutional-recurrent essay scoring model is the *hierarchical* approach to representing student texts by Dong et al. (2017). Notably, both Alikaniotis et al. (2016) and Taghipour and Ng (2016) processed student texts strictly on the *word* level, reading in each input text as a sequence of word tokens without any explicit modelling of any other units within a given text, e.g. on the sentence level. In contrast, Dong et al. (2017) first used a CNN to obtain sentence representations out of word representations, and then fed the sentence representations into an LSTM to produce final essay representations for score prediction. This architecture is depicted in Fig. 4, where each instance of **x**_1...*n*_ at the model input level represents a *sentence*, i.e. a sequence consisting of *n* word tokens.

Unlike Taghipour and Ng (2016), Dong et al. (2017) found attention pooling to outperform average pooling and used it both on the sentence-level and the essay-level representations. They suggest that their hierarchical model architecture encouraged the positive effects of attention pooling across the LSTM outputs. Specifically, their work argues that since the input sequences to the LSTM were sequences of sentence representations instead of word representations, they were significantly shorter, which allowed attention pooling to be more effective.

##### Word and Character Embeddings

At the model input level, student texts fed to both RNNs and CNNs are typically represented as sequences of word-level vector representations, known as *word embeddings*. Word embeddings represent words in terms of their distributional context. They can be separately pre-trained on language modelling tasks using large unlabelled corpora and repeatedly reused as a look-up dictionary mapping each in-vocabulary token to its corresponding vector representation. Word2vec (Mikolov et al., 2013; Mikolov et al., 2013) and GloVe (Pennington et al., 2014) are among the most popular openly available resources for obtaining pre-trained word embeddings for English and have been been used in numerous neural approaches to student free-text evaluation, including recent ones (Dong et al., 2017; Riordan et al., 2017; Kumar et al., 2020).

As an extension to word-level embeddings, (Bojanowski et al., 2017) proposed encoding subword-level information into word embeddings. That is, they trained embeddings for *character* n-grams, i.e. character strings of length *n* that constitute words, and took the sum of words’ constituent character n-gram embeddings to be their word embeddings. In the educational domain, models incorporating character embeddings have been shown to be more robust against spelling errors in students’ texts (Horbach et al., 2017) because character embeddings capture the relatedness between a word, e.g. *information*, and its misspelled counterpart e.g. *infromation*, with which it shares many substrings. However, the benefits of character-level embeddings for addressing misspellings in student or language learner texts are inconclusive; Riordan et al. (2019) found in their studies that while they did show positive effects, they were not as effective as performing spelling correction on the training data as a pre-processing step.

#### Pre-Trained BERT for Student Free-Text Evaluation

Traditional pre-trained word embeddings such as GloVe (Pennington et al., 2014) map each word token to a single *context-insensitive* vector representation, which means that the same vector is used for all senses of an ambiguous word like *port* in English.^7^ This is evidently not optimal and has motivated the development of large pre-trained language models that generate deep *contextualised* word representations for each word dependent on the individual linguistic context in which it occurs (Peters et al., 2018; Devlin et al., 2019).

BERT (Bidirectional Encoder Representations from Transformers) (Devlin et al., 2019), in particular, has become tremendously popular in a wide variety of NLP tasks (Sun et al., 2019; Yang et al., 2019). We refer to Devlin et al. (2019)’s original paper for a detailed description of BERT. In brief, it is a large transformer-based language model trained on tremendous amounts of unlabelled data. It takes as input a token sequence consisting of two segments of text in which some tokens are masked. The two segments are joined by a special separator token [SEP], and the full sequence is prepended by the special token [CLS]. During training, BERT simultaneously learns to perform two tasks: Predicting the masked tokens based on the known tokens in the input (masked language modelling (MLM)), and predicting whether the second text segment truly follows the first segment in the training corpus (next sentence prediction (NSP)). To do so, the model learns deep representations for each input token, based on which MLM is performed; the representation of the special [CLS] token is learned as a representation of the whole input sequence and used to perform NSP.

The common, fine-tuning-based approach to using BERT consists of pre-training it on the language modelling tasks mentioned above using unlabelled data and transferring the full pre-trained model to a target task of interest, where the model is fine-tuned on a dataset labelled for the target task (Devlin et al., 2019). A wide variety of NLP-related target tasks have been shown by Devlin et al. (2019) to benefit from this usage of BERT.

**Fig. 5:**
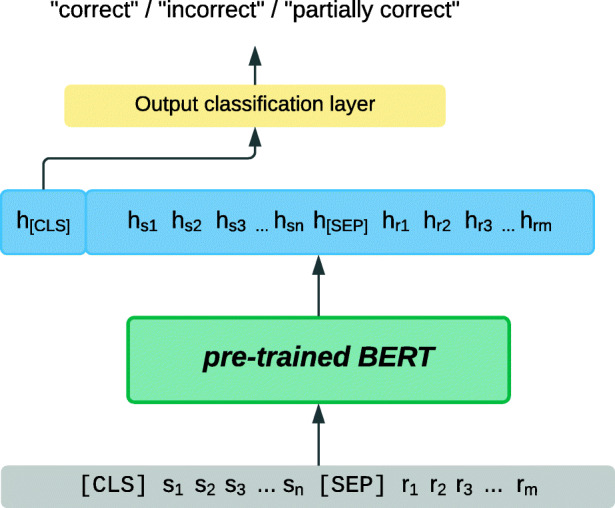
Short-answer scoring model based on fine-tuning a pre-trained BERT model, adapted from Sung et al. (2019); variables are explained in the text

In the field of student free-text evaluation, Sung et al. (2019) provided a representative and simple method of using pre-trained BERT for scoring students’ short answers against reference answers: Given pairs of student answers and reference answers to some question, the task was to automatically classify the student answers as *correct*, *incorrect* or an additional class such as *partially correct*. In Sung et al. (2019), a pre-trained BERT model was fine-tuned on pairs of student and reference answer sequences, prepended by the special [CLS] token. It learned to classify the student answers based on its output representation for the [CLS] token, which, as mentioned, was learned as a representation of the whole input sequence. Figure 5 shows this fine-tuning step, where **s** and **r** denote student and reference answers, which are token sequences of lengths *n* and *m*, respectively, and **h** denotes the corresponding contextualised representation of each input token.

Apart from the fine-tuning approach, Devlin et al. (2019)’s original paper also proposes a *feature-based* approach as an alternative method of using BERT: In this scenario, the pre-trained BERT model is frozen. During training on a specific target task, pre-trained BERT is fed input data of the target task and generates contextualised representations of the input data. These representations are then extracted out of BERT and used to initialise the input layers of a *separate* task-specific model in the same manner as using traditional word embeddings like GloVe to initialise neural models. In this case, the pre-trained and static BERT model only acts as an extractor of contextualised embedding features and does not fine-tune itself on the target task data.

This feature-based approach to using BERT has also been exploited in student free-text evaluation: As a component of their essay scoring model, Liu et al. (2019) used BERT to extract dense representations for each sentence in their input essays. They did so by performing average pooling over the contextualised word embeddings that were generated by pre-trained BERT for all the words in the sentence. Sentence embeddings obtained in this manner were then fed as input representations to a separate LSTM to produce representations of full essays. Similarly, Nadeem et al. (2019) used contextualised word embeddings produced by a static BERT model to initialise their LSTM-based essay scoring architecture.

### Combination of Neural and Feature-based Models

The previous two sections outlined approaches to student free-text evaluation using manually engineered features on the one hand and neural models and features on the other. However, hybrid approaches combining the two are common and successful as well.

The essay scoring model by Liu et al. (2019), mentioned above, used BERT-embeddings fed to an LSTM to obtain a representation and an intermediate semantic score of student essays. Two additional LSTMs were separately trained to specifically model and score the coherence of the essay and the extent to which a student essay matches the essay prompt. At a second stage, they added a set of manual features similar to those described in “Feature Sets and Models”, including the number of linguistic errors and length-based features. The full feature vector combining the neural scoring features and the manual features were then fed to a gradient boosting decision tree for the prediction of the final essay score.

**Fig. 6:**
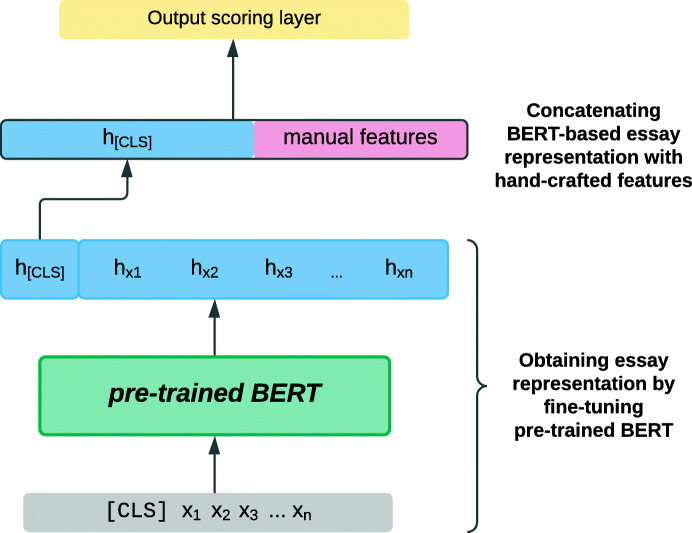
Hybrid essay scoring model based on fine-tuning a pre-trained BERT model and concatenating the resulting essay representation with an essay vector consisting of hand-crafted features, adapted from Uto et al. (2020); variables are explained in the text

Uto et al. (2020) offered a simpler and effective method for combining neural and hand-crafted features: Neural models ultimately compute a dense vector representation of a given input text in order to score it. Uto et al. (2020) propose concatenating the deep representation with hand-crafted linguistic feature vectors and feeding the composite vector to an output layer for score prediction. That is, in their approach, manual features were injected into a neural architecture at the pre-output layer. To illustrate, Fig. 6 shows the architecture of this hybrid approach, in which the neural essay representation is obtained by fine-tuning a pre-trained BERT model; **x** denotes input tokens that form a sequence of length *n*, and **h** denotes their contextualised embeddings.

Uto et al. (2020) demonstrated the benefits of hybrid approaches to essay scoring: They experimented with different neural architectures for deriving the neural essay representations, including BERT and LSTMs. In each case, they contrasted the scoring performance using the neural representation *alone* versus performance based on the same representation *concatenated with additional manual features*. When hand-crafted features were added, they observed significant performance increases.

Combinations of neural and feature-based approaches to student free-text evaluation are particularly attractive for several reasons: First, while neural approaches generate representations on the word or even subword level and go from there to building representations of full texts, hand-crafted features such as average sentence length or lexical diversity can capture characteristics on the essay or document level. The two approaches can be considered complementary (Uto et al., 2020), which could explain their success in the works mentioned above. Second, hybrid approaches can leverage both the increased expressive power of neural models and insights from decades of essay assessment research that has identified informative linguistic features (Shermis and Burstein, 2013). Finally, neural text evaluation models, in particular, require large amounts of training data; Nadeem et al. (2019) found that particularly where labelled training data is limited, adding hand-crafted features improve the performance of neural models.

## Recent Work on Student Free-Text Evaluation

In this section we provide an overview of the latest representative work on student free-text evaluation, most of which make use of the ML techniques discussed in the previous section. We consider automatic essay evaluation (AEE), both in terms of holistic essay scoring and evaluation along specific aspects of writing,^8^ and automatic short-answer scoring (ASAS). As previously noted, targeted, formative feedback to students’ performance in e-learning environments presupposes accurate evaluation of the students’ input; therefore, AEE and ASAS are of immense interest to the development of intelligent tutoring systems.

For reasons of space, our survey excludes works that specifically target grammatical error correction in essays, which is extensively treated in its own body of literature (see, for instance, Bryant et al. (2019)). Areas that we also consider to be beyond the scope of this survey include the assessment of students’ written reflection on their own study (e.g. Carpenter et al. (2020)), which is a very specific genre of student texts, and students’ interaction data from computer-supported collaborative learning (e.g. Trausan-Matu et al. (2014)), which does not deal with the evaluation of an individual students’ performance.

To give an easily accessible overview, Table 1 lists the recent works that will be presented more thoroughly in the remainder of this section. We also include Sung et al. (2019), Liu et al. (2019) and Uto et al. (2020), which have been discussed in detail in the previous section.

**Table 1:** Overview of recent works presented in this section; more details are given in the text

Work	Relevant Contributions
Holistic Essay Scoring	
Liu et al. (2019)	Scoring in English with modelling of coherence and prompt appropriateness; two-stage hybrid approach
Uto et al. (2020)	Scoring in English; hybrid approach by concatenating manual and neural features at pre-output level
Xue et al. (2021)	Scoring in English, both holistic and individual traits; BERT-based MTL model that jointly trains on all topics
Nadeem et al. (2019)	Scoring of English argumentative essays, using cross-sentence attention to capture discourse coherence
Yang and Zhong (2021)	Scoring in English; coherence modelling via similarity between adjacent sentences; topic relevance modelling via similarity with essay prompt
Zhang and Litman (2018)	Scoring English source-based essays; using attention to capture relation between essay and source text
Horbach et al. (2017)	Scoring German essays, both holistic and individual traits
Song et al. (2020)	Scoring Chinese essays; pre-training on scoring essays on other prompts before final fine-tuning on essays on target prompts
Evaluation of Specific Aspects of Essays	
Mathias and Bhattacharyya (2020)	Scoring individual traits in English essays
Hellman et al. (2020)	Scoring topic-specific content in English essays; multiple-instance approach assigns scores to individual sentences and an aggregated overall score
Ghosh et al. (2016)	Scoring the argumentation aspect in English persuasive essays using argumentation features
Alhindi and Ghosh (2021)	Argumentation evaluation for English essays by using BERT-based model to detect argument components
Wambsganss et al. (2020)	Feature-based argumentation structure assessment in German essays for formative feedback generation
Šnajder et al. (2019)	Evaluation of discourse structure in English summary essays by extracting rhetorical relations from student essays and comparing them with those found in experts’ summaries
Song et al. (2020)	Evaluation of discourse structure in Chinese and English argumentative essays by extracting discourse elements
Song et al. (2020)	Evaluation of essay organisation in Chinese essays via hierarchical representations of sentences, paragraphs and whole essays
Zhang et al. (2019)	Evaluation of students’ use of information from the source text in source-based English essays, targeting feedback generation
Short-Answer Evaluation Without Using Reference Answers	
Riordan et al. (2017)	Scoring short answers in English; among the first to apply neural essay scoring techniques to ASAS
Riordan et al. (2019)	GRU-based approach to ASAS in English incorporating spellchecking and character-level information
Riordan et al. (2020)	BERT-based approach to ASAS in English along specified rubrics laid down by educational authorities
Kumar et al. (2020)	Feature-based model to ASAS in English using both linguistically motivated features and embedding features
Cahill et al. (2020)	Scoring English short answers that contain mathematical expressions; regular expressions used first to extract mathematical expressions
Mizumoto et al. (2019)	ASAS model for Japanese that predicts holistic and rubric-based scores, justifying its decision by highlighting relevant parts in the answers
Ding et al. (2020)	Highlighting the vulnerability of feature-based and neural ASAS models to adversarial input that imitate gaming attempts
Short-Answer Evaluation Based on Reference Answers	
Maharjan and Rus (2019)	Evaluation of English student answers to Physics topics by using concept map representations for both student and reference answers
Sung et al. (2019)	Among the first to apply a BERT-based approach to reference-based ASAS for English
Li et al. (2021)	ASAS for English; using relation network to capture relations between student answers, references and question prompts

### Automatic Essay Evaluation (AEE)

AEE or essay scoring has a fairly long history going back to the Project Essay Grade in the 60s (Page, 1966). Since then, it has continued to be an area of much active research (Shermis and Burstein, 2003; Attali & Burstein, 2006; Shermis & Burstein, 2013; Uto, 2021). Work is ongoing for assessing a wide range of classes of essays, including essays by middle school students (Zhang et al., 2019) and by university students (Hellman et al., 2020), by native (Uto et al., 2020) and non-native speakers (Ghosh et al., 2016), as well as different genres of essays, e.g. summary essays (Šnajder et al., 2019) and persuasive essays (Nguyen & Litman, 2018). In the following sections, we first look at recent work targeting the *holistic scoring* of student essays, then move on to approaches that evaluate specific aspects of essays.

#### Holistic Essay Grading

Latest works that are based on fine-tuning pre-trained BERT models to perform holistic essay scoring include Xue et al. (2021). Instead of feeding in complete essays as input to BERT, they split the essays into multiple fractions and computed BERT-based deep representations for each fraction. Attention pooling was then applied to the fraction representations to obtain single representations of full essays for scoring. This approach proved to improve performance on long essays. Furthermore, while essay scoring models can be individually trained and tested on topic-specific data, Xue et al. (2021) used a multi-task learning (MTL) approach. Their MTL-model was trained on data covering multiple topics and jointly learned to score essays on all topics. They found in their experiments that training a single model on a large, multi-topic dataset outperformed separate models trained on smaller, topic-specific datasets.

**Fig. 7:**
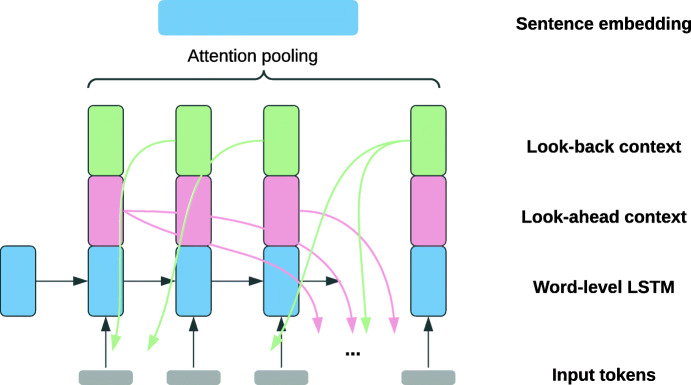
Computation of sentence-level embeddings from token-level representations using the cross-sentence dependencies described in Nadeem et al. (2019)

Various works modelled discourse-level properties of essays in order to improve overall essay scoring. Examples include Nadeem et al. (2019). They used hierarchical LSTMs with attention pooling to compute sentence and essay representations. To this basis, they added *cross-sentence dependencies*: Before applying attention pooling across the hidden outputs of the word-level LSTM to yield sentence embeddings, they concatenated each token’s hidden output with a *look-back* and *look-ahead* context vector, where the context vectors were designed to capture the similarities of the token with each token in the preceding and the following sentence (see Fig. 7 for a visualisation). The token-level hidden outputs that were enriched in this manner with cross-sentence context were then aggregated to obtain sentence-level embeddings. Similarly, Yang and Zhong (2021) modelled coherence by computing the similarities between individual sentences within essays. They also captured essays’ relevance to the expected essay topic based on similarities between essay sentences and the prompts.

Student essays can be *source-dependent* in that they are written in response or in reference to some reading source material. For instance, in the following prompt (taken from the ASAP++ dataset (Mathias and Bhattacharyya, 2018)), students are asked to read an excerpt from a memoir by designer Narciso Rodriguez. Then they are given the following task prompt (reproduced from (Zhang & Litman, 2018, p. 3)):

**Essay Prompt:** Describe the mood created by the author in the memoir. Support your answer with relevant and specific information from the memoir

Zhang and Litman (2018) specifically targeted source-dependent student writings and incorporated the source text in their models. They used *co-attention* to enrich the representation of the essays with information regarding their relation to the source text, which proved to have positive effects on the scoring of source-dependent essays in their studies. Concretely, they applied Dong et al. (2017)’s architecture to encode the student essay on the one hand and the source text on the other. At the level of sentence representations, they used attention across the student and source texts to capture, for each sentence in the student essay, which sentence in the source text it was most similar to. These enhanced representations of essay sentences were then fed to an LSTM for producing the final student essay representation.

All of the work presented above have targeted scoring essays *in English*. Work on non-English text is scarce. A rare example of that is (Horbach et al., 2017), who worked on essays written in German by university students (native speakers), predicting both holistic scores and scores for different evaluation rubrics. They experimented with both the neural model by Taghipour and Ng (2016) and an SVM using n-gram features and found that their task posed significant challenges to either model. They attribute this both to the German language and to the general high level of writing proficiency demonstrated in the essays.

Song et al. (2020) scored middle and high school essays in Chinese and used multiple stages of pre-training and transfer learning. Their target task was the holistic scoring of essays with a particular prompt. They used Dong et al. (2017)’s model architecture and pre-trained the model first on an unrelated set of essays with coarse ratings, then on labelled essays of the same type as the target essays but with *different* prompts, before finally fine-tuning on the target set of essays. While successful, this approach of course presupposed the existence of labelled data for each of the pre-training and fine-tuning tasks.

Finally, a number of work on non-English data have targeted essays written by non-native speakers learning the respective language. However, the task would often be the prediction of the learner’s proficiency level based on the essay rather than evaluation of the essay itself. A recent example is Johan Berggren et al. (2019) for Norwegian, who experimented with both feature-based and neural models and obtained their best results using a bidirectional GRU architecture. Earlier work includes (Pilán et al., 2016) for Swedish and (Vajjala and Loo, 2014) for Estonian.

#### Evaluation of Aspects of Student Essays

Instead of giving a holistic score, a separate body of work have dealt with models for the scoring or evaluation of specific aspects or traits of essays, which can be better suited for providing formative feedback. Mathias and Bhattacharyya (2020) predicted individual scores for specific essay traits including content, word choice, sentence fluency, writing conventions etc. They obtained trait-specific scores for all essays in their dataset and trained the hierarchical model by Dong et al. (2017) for each essay trait individually. Xue et al. (2021) also labelled their essays with individual scores for various essay traits, but their model jointly learned to score all of the essay traits in an MTL fashion.

The approach by Hellman et al. (2020) to content-specific essay scoring is particularly noteworthy: In their task formulation, given a student essay and a set of expected content topics that the student essay is expected to cover, the model would give a score with regard to each topic and indicate how well the student essay covers that specific topic. They approached this task with multiple instance learning (MIL), in which they used the k-nearest-neighbour algorithm to give a score to each sentence within the essay with respect to a topic; the topic-specific score for the whole essay was then an aggregation of the topic-specific scores for each sentence. Crucially, since they obtained sentence-level scores with respect to a specific topic, they could give fine-grained feedback about students’ treatment of that topic by pointing to very specific parts in their essay.

Ghosh et al. (2016) and Nguyen and Litman (2018) scored persuasive essays by modelling the argumentation structure in the essays. Both used argumentation features in addition to baseline essay scoring features such as length-based features and found the addition useful. Argumentation features pertain to the *argumentative structure* in a persuasive text and can be automatically extracted via argumentation mining techniques. Following established approaches (Stab & Gurevych, 2014; Peldszus & Stede, 2016; Stab & Gurevych, 2017), argumentation mining recognises argumentative components and their relations in texts. For instance, in the following example from (Stab & Gurevych, 2017, p. 628), a so-called *claim* in favour of cloning (in bold) is identified as being supported by a so-called *premise* (in italics):

First, **cloning will be beneficial for many people who are in need of organ transplants**. *Cloned organs will match perfectly to the blood group and tissues of patients*.

Argumentation features which can be exploited in a feature-based ML approach to essay scoring include the number of the different argument components and relations etc. (Ghosh et al., 2016).

Other than providing features for scoring, the evaluation of students’ argumentation behaviour can be interesting in its own right as the basis for feedback. Alhindi and Ghosh (2021) performed recognition of argument components in middle school students’ essays based on recent neural models including BERT. Wambsganss et al. (2020) presented a feature-based argument mining system using linguistic features and traditional classifiers such as SVM. They analysed the argumentation structure in essays by German-language business students and provided feedback to students on their argumentation skills in a dashboard. Related work has also been done for language learner essays: Putra et al. (2021) experimented with neural approaches to argument mining on college-level essays by non-native English speakers from Asian countries.

While argumentation is mostly relevant to persuasive essays, discourse structure and organisation are general components indicating essay quality. Šnajder et al. (2019) evaluated the rhetorical structure of students’ summaries written in response to a source text and rated them against reference summaries. They used an off-the-shelf discourse parser to extract the rhetorical relations from student and reference summaries and rated the amount of matches using semantic similarity measures. Song et al. (2020) evaluated the discourse structure in students’ argumentative essays in Chinese and English, which they cast as a sentence-level classification task where the class labels were discourse elements such as *introduction*, *conclusion* etc. They used an LSTM-based model and found it useful to encode the position of each sentence in the essay as well as to incorporate attention across sentences. Another state-of-the-art neural model is the MTL model for evaluating the organisation of student essays by Song et al. (2020). They cast the overall task as a combination of three tasks that were jointly trained: the classification of each sentence to a set of sentence functions, the classification of each paragraph to a set of paragraph functions, and the evaluation of the overall essay organisation in terms of a coarse-grained rating. This was achieved by hierarchically building dense vector representations of sentences, paragraphs and finally essays, where a linear layer was added to each representation level for classification.

Content-oriented evaluation for subsequent feedback was the focus of the eRevise system (Zhang et al., 2019) for writing support in source-dependent essays. In their use case, middle school students read an article and were asked to voice their positions on the topic addressed in the article. The eRevise system specifically evaluated how well students had referred to and made use of evidence from the source text and gave feedback accordingly. They used a sliding window to extract items in the student texts that corresponded to key topics from the article, using lexical similarity measures to account for synonyms. While in the early version of eRevise, such key topics (referred to as *topical components*) were manually created for each source article, the authors have since worked on automatically extracting them (Zhang and Litman, 2020; 2021). The emphasis of eRevise was put on providing relevant feedback to the writer. To illustrate, where the system detected little usage of evidence from the source text, the feedback message could be *Re-read the article and the writing prompt*; if good usage of source text evidence was found, the feedback could be more specific, such as *Tie the evidence not only to the point you are making within a paragraph, but to your overall argument* (Zhang et al., 2019, p. 9621).

### Automatic Short-Answer Scoring (ASAS)

ASAS is related to AEE but deals with the evaluation of students’ significantly shorter free-text answers to question prompts. Riordan et al. (2017) has remarked on some noteworthy differences between the two: Unlike AEE, where writing skills as expressed by style, structure etc. play a role, ASAS typically focuses exclusively on the correctness of *content*. Furthermore, while AEE is frequent in language classes, ASAS is more commonly applied to mathematics and science topics. The following shows an example from the Student Response Analysis dataset (Dzikovska et al., 2012), which consists of the question prompt, a reference answer and an example of a correct and an incorrect candidate student answer (reproduced from (Riordan et al., 2017, p. 161)):

**Prompt**: What are the conditions that are required to make a bulb light up **Reference answer**: The bulb and the battery are in a closed path **Student answer**:

- **correct**: a complete circuit of electricity
- **incorrect**: connection to a battery

ASAS is challenging because answers expressing the same content, whether correct or incorrect, can be linguistically expressed in vastly different ways (Horbach and Zesch, 2019). In the example above, the correct student answer is correct despite the complete lack of vocabulary overlap with the reference answer, while the incorrect one shares the term *battery* with the reference but is nonetheless incorrect.

We present two broader groups of recent approaches to ASAS. In the first, scoring is performed without explicit usage of any reference answers, whereas in the second, student answers are evaluated against a reference. This is what Horbach and Zesch (2019) have referred to as *instance-based* versus *similarity-based* approaches.

#### ASAS without Reference Answers

In the absence of reference answers, the set-up in ASAS in the most straight-forward form is predicting a score, given a piece of textual input. As such, the same approaches for essay scoring can be applied: Riordan et al. (2017) experimented with applying the convolutional recurrent essay scoring model by Taghipour and Ng (2016) to ASAS. They found that the model had successfully transferred to short-answer scoring, although they found that tuning hyper-parameters specifically to ASAS and applying alternative pooling methods improved performance. In subsequent work, Riordan et al. (2019) used a similar neural architecture based on GRUs, adding character-level representation as well as spell-checking as a pre-processing step, and obtained competitive results. Targeting middle school science classes, Riordan et al. (2020) experimented with various models including feature-based SVM, recurrent and BERT-based models to score students answers according to specific rubrics laid down by educational authorities. They found the approach based on fine-tuning BERT to be particularly successful.

Kumar et al. (2020) used a random forest feature-based model to score ASAS. Their feature set included a spectrum of linguistic features, including part-of-speech, weighted keywords, logical operators, lexical diversity etc., to name just a few. Moreover, they also included the pre-trained classical embeddings from word2vec (Mikolov et al., 2013) and doc2vec (Le and Mikolov, 2014) as features. Aside from achieving highly competitive results, they conducted a feature ablation study which revealed the top predictors to be the embedding features, weighted keywords, and the lexical overlap with question prompts.

A particularly noteworthy piece of work is Cahill et al. (2020)’s approach to scoring short-answers to complex mathematical questions that contain both natural language and mathematical expressions. To illustrate, an extract from a student’s short-answer provided by the authors is shown below (Cahill et al., 2020, p. 187):

$x = \frac {-40 + \sqrt {40^{2}-4(-2)(-195)}}{2(-2)}$To solve this you must first put your equation in standard form, which gives you *y* = − 2*x* + 40*x* − 195. You then plug your *a*, *b*, and *c* values into the quadratic formula. To start finding your *x*, you must first multiply all your values in parentheses. You must then simplify the square root you get from multiplying [...]

The authors’ approach started by using regular expressions to recognise mathematical expressions. These purely formulaic expressions were sent to a separate tool for evaluation as correct or incorrect. Special tokens that indicated mathematical expressions as well as their correctness were then used to replace the actual expressions in the text. For instance, the first sentence in the above example answer could be converted to *To solve this you must first put your equation in standard form, which gives you @correct@*, where *@correct@* would denote the presence of a mathematical expression that had been evaluated as correct. Finally, the resulting text, which was then free from mathematical expressions, was sent to various text scoring regression models. Cahill et al. (2020) obtained strong results from a GRU-based model as well as an SVM using the special tokens with mathematical information as features.

**Fig. 8:**
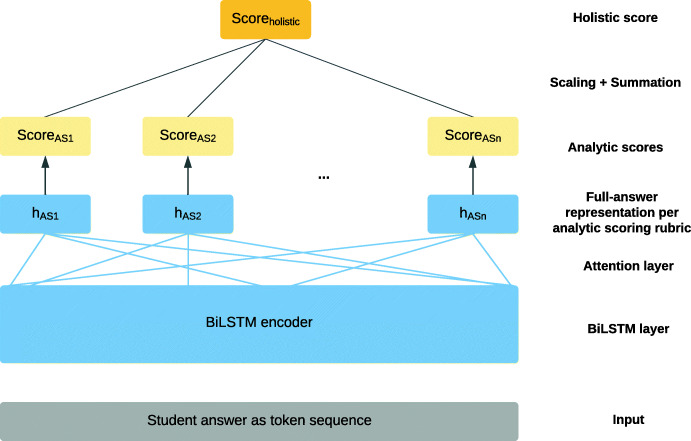
Model for jointly predicting analytical and holistic scores for student short-answers, adapted from Mizumoto et al. (2019); variables are explained in the text

One of the few recent works on non-English data is research by Mizumoto et al. (2019) on Japanese short-answer scoring. Their model notably incorporated methods for pointing students to specific parts in their answer to explain the score given, which they term *justification identification*. This is reminiscent of Hellman et al. (2020) for essay scoring (see above). In the task by Mizumoto et al. (2019), for each student response, both a holistic score and a set of so-called *analytic scores* were predicted, where each analytic score addressed a specific scoring rubric related to the specific prompt. Inspired by Riordan et al. (2017), they used BiLSTM-based neural models with attention pooling to generate representations of the full student answer. Notably, for each analytic score, a distinct score prediction model was trained by taking the BiLSTM outputs and computing an attention vector *specific to that analytic score*. A representation of the full answer *with respect to that analytic score* was then obtained by attention pooling across the BiLSTM outputs using the attention weights for that analytic score. Subsequently, the short-answer representations specific to each analytic score were then each sent to a linear prediction layer. Finally, the predicted analytic scores were scaled and summed to produce the predicted holistic score. This architecture is illustrated in Fig. 8, in which *AS* stands for *analytic score*, **h**_*AS*_ denotes a representation of the full student answer for each of *n* analytical scoring rubrics, and *score*_*AS*_ denotes the predicted analytical score for each rubric. Computing a distinct representation of the full short-answer with respect to each analytic score captured the fact that each analytic score addressed a distinct scoring rubric and would be determined by different parts in the student answer.

For justification identification with respect to each analytic scoring rubric, Mizumoto et al. (2019) made use of the respective attention weights in the attention pooling step of the models, which would indicate which parts of the student answer the model had attended to when producing a specific analytic score. This information was then presented to students to justify the score. The following example illustrates justification identification in a student answer with respect to two analytic scoring rubrics (reproduced from (Mizumoto et al., 2019, p. 316) and simplified):

**Prompt**: Explain what the author means by the phrase ”this tension has caused several different philosophical viewpoints in Western culture” **Student Answer**: Conflicts of interest in *Western culture* are formmed[sic] on the basis of God vs. Human.

- **Analytic scoring rubric A** (see italicised parts in student answer): Mentions “Western culture” or “Western”
- **Analytic scoring rubric B** (see underlined parts in student answer): Mentions “others have different view points from oneself”

The analytic scoring rubric B deals with the notion of people having different viewpoints. Since the student answer correctly addresses this notion, the well-performing scoring system would produce a high analytic score for rubric B. The attention weights used for computing the student answer representation specific to rubric B would reveal large weights for the BiLSTM outputs for the tokens *conflicts*, *of* and *interest*, which would show these tokens to be decisive for the analytic score prediction for rubric B.

Ding et al. (2020)’s work on scoring *adversarial* short-answers highlight an important challenge in ASAS: Models tend to be trained to recognise correct answers despite orthographic errors and to be robust to various levels of variance in student answers (Horbach and Zesch, 2019), e.g. by incorporating character-level representation (Riordan et al., 2019). However, they should also be robust to potential gaming and cheating attempts and reject wrong answers that are made to resemble correct answers. In their experiments, Ding et al. (2020) artificially generated a series of adversarial short-answers to prompts from the popular dataset from the Automated Student Assessment Prize^9^ (ASAP). The answers were generated to resemble possible gaming attempts by students. These adversarial samples included random character or word sequences, random content words related to key words in the prompt, shuffled tokens from real correct answers etc. To illustrate, in response to a prompt that asked for a comparison between pandas, koalas and pythons, the authors provided the following examples of adversarial answers, among others (Ding et al., 2020, p. 884):

**Random characters**: fcwowtpmqalwkjxldrldvc bw fhgkter **Random words**: footage flubbed birthplace parry’s cicadas **Content words related to prompt**: panda eat bamboo koala eucalyptus python America need fact comparison resource people **Token shuffling of correct answers**: bamboo eucalyptus resources eats koalas need eat anything panda but and as doesn’t the America...

Ding et al. (2020) then trained both an SVM with word and character n-grams and the neural system by Riordan et al. (2019) on the official ASAP training data and tested them on their generated adversarial answers. Their findings revealed that both systems, in particular the neural one, were highly vulnerable to such adversarial input, with the neural system accepting nearly half of the adversarial answers as at least partially correct. The authors found that training on adversarial data helped to alleviate the problem but nonetheless did not solve it, which suggests that adversarial answers that might represent cheating attempts remain a major challenge.

#### ASAS based on Reference Answers

In scenarios where student answers are explicitly assessed against a reference answer, models process a *pair* of text as input. “Pre-Trained BERT for Student Free-Text Evaluation” has already presented the BERT-based approach by Sung et al. (2019).

A novel approach to ASAS on physics topics has been proposed by Maharjan and Rus (2019). While their system compared student responses with reference answers, the comparison was not done on the textual level, but on the level of *concept map* representations. Concept maps are graphical knowledge representations consisting of knowledge triplets, where each triplet comprises two concepts and the relation between them. An example triplet given by the authors is (velocity, be, constant) for the sentence *velocity is constant*. Maharjan and Rus (2019) obtained concept maps for reference answers; at run time, they extracted such knowledge triplets from student responses using available tools for information retrieval. This approach not only allowed the system to evaluate the correctness of students’ responses but also provided a straightforward way to identify missing triplets in the student answers and to give feedback on them.

While a common approach to reference-based answer scoring models the similarity between student and reference answers (Sung et al., 2019; Maharjan & Rus, 2019), Li et al. (2021) used a Semantic Feature-Wise transformation Relation Network (SFRN) to encode the general relation that held between a question (Q), a student answer (S) and all applicable reference answers (R). The resulting representation of a given QSR-triplet was then fed to a scorer. Their approach can also be applied to datasets that do not come with reference answers but do provide grading rubrics. In that case scoring would be performed by encoding the relation between triplets of questions, student answers and scoring rubrics.

## State-of-the-Art on Popular Datasets

This section presents some of the most frequently used datasets for essay and short-answer scoring and state-of-the-art results reported on them. We explicitly do not aim to provide a comprehensive list of available datasets^10^ but limit our discussion to datasets that have been widely used in recent work.

### Essay Scoring

By far the most common dataset on which essay scoring results have been reported is the English-language data released in 2012 by Kaggle as part the Automatic Student Assessment Prize (ASAP), sponsored by the Hewlett Foundation. They have provided an openly available dataset for essay scoring, ASAP-AES^11^ and one for short-answer scoring ASAP-SAS^12^. ASAP data is used in 90% of the English-language essay and short-answer scoring systems examined by Ramesh and Sanampudi (2021).

ASAP-AES comprises approximately 13,000 essays, written in response to 8 prompts. It includes narrative, argumentative and source-dependent essays written by US school students in grades 7-10. Holistic scores are provided for each essay, although the score range varies across prompts. Shermis and Burstein (2013) and Mathias and Bhattacharyya (2018) offer detailed descriptions of ASAP-AES.

Numerous work presented in “Automatic Essay Evaluation (AEE)” train and evaluate their essay scoring systems on ASAP-AES, including

- Alikaniotis et al. (2016)
- Taghipour and Ng (2016)
- Dong et al. (2017)
- Nguyen and Litman (2018)
- Zhang and Litman (2018)
- Liu et al. (2019)
- Nadeem et al. (2019)
- Mathias and Bhattacharyya (2020)^13^
- Uto et al. (2020)
- Yang and Zhong (2021)
- Xue et al. (2021)

The official evaluation metric used by the ASAP competition and therefore adopted by most work is the *quadratic weighted kappa* (QWK), which measures the amount of agreement between two annotators, in this case the model prediction and the gold-label score.^14^ In Table 2 we summarise the reported average QWK scores across all 8 prompts by some of the recent systems. Works that do not evaluate on all of the 8 prompts (Nguyen and Litman, 2018; Nadeem et al., 2019; Zhang & Litman, 2018) are not included. We also exclude Alikaniotis et al. (2016) since they do not evaluate with QWK.

**Table 2:** Mean QWK results on ASAP-AES achieved by various paper’s respective best system, with the best result in bold

System	Mean QWK Across All Prompts
Taghipour and Ng (2016)	0.761
Dong et al. (2017)	0.764
Liu et al. (2019)	0.773
Yang and Zhong (2021)	0.788
Uto et al. (2020)	0.801
Xue et al. (2021)	**0.830**

To the best of our knowledge, the current state-of-the-art on the full ASAP-AES dataset has been achieved by Xue et al. (2021)’s BERT-based MTL system, which jointly trained on ASAP-AES data for all topics in an MTL fashion. Competitive results have also been achieved by the hybrid system by Uto et al. (2020).

Aside from ASAP-AES, which dominates the field, another dataset repeatedly used for essay scoring is the ETS TOEFL11 dataset released by the Linguistic Data Consortium (LDC)^15^ (Blanchard et al., 2013). Originally collected with the task of native language identification in mind, the corpus consists of over 12,000 essays by university-level non-native speakers written as part of the TOEFL exam. Like ASAP-AES, essays cover 8 writing prompts and various essay types including narrative and argumentative essays. Holistic scoring is provided on a 3-point rating system of *Low*, *Medium* and *High* (see Blanchard et al. (2013) for details).

Recent essay scoring work using TOEFL11 include

- Ghosh et al. (2016)
- Nguyen and Litman (2018)
- Nadeem et al. (2019)

Comparison between systems on the TOEFL11 data is difficult since different authors have used different subsets of the corpus: Ghosh et al. (2016) used only a selection of 107 argumentative essays, whereas Nguyen and Litman (2018) used a subset of over 8000 essays. Among the work listed above, only Nadeem et al. (2019) used the full TOEFL11 set. They reported their best rating result as a QWK of **0.729**, obtained by their neural model with BERT embedding features and cross-sentence dependencies (see “Automatic Essay Evaluation (AEE)”).

General essay scoring datasets for languages other than English are rare, and we are not aware of benchmark datasets that have been reported on by multiple works. Horbach et al. (2017) have compiled a corpus for holistic and trait-specific essay scoring on German from university students, and Östling et al. (2013) have collected an essay scoring dataset for Swedish from national high school examinations; however, to our knowledge, neither dataset is publicly available due to legal restrictions. Various work has been done on Chinese essays (Song et al., 2020; Song et al., 2020; Song et al., 2020), but in each case the authors perform their own data collection dedicated to their tasks.

### Short-Answer Scoring

For short-answer scoring, the dataset most commonly reported on is again the Kaggle ASAP dataset, i.e. ASAP-SAS. The dataset consists of over 16,000 responses to 10 question prompts from a wide range of subject areas, including science and reading comprehension. The responses are obtained from US high school students and scored holistically. No reference answers are used, but scoring rubrics are available. Further details on the dataset are provided by Shermis (2015). Recent studies on ASAP-SAS include:

- Riordan et al. (2017)
- Riordan et al. (2019)
- Kumar et al. (2020)
- Li et al. (2021)

Evaluation for ASAP-SAS is once again the QWK measure. Table 3 shows the mean QWK results of the above systems on ASAP-SAS. To our knowledge, the current state-of-the-art has been achieved by Kumar et al. (2020), who combined a feature-based model with static neural embeddings, and by the latest SFRN-model by Li et al. (2021).

**Table 3:** Mean QWK results on ASAP-SAS achieved by various paper’s respective best system, with the best result in bold

System	Mean QWK Across All Prompts
Riordan et al. (2017)	0.732
Riordan et al. (2019)	0.779
Kumar et al. (2020)	**0.791**
Li et al. (2021)	**0.79**

Among the most popular datasets for reference-based short-answer scoring is the Student Response Analysis (SRA) dataset (Dzikovska et al., 2012), which was prominently used in the SemEval 2013 shared task *The Joint Student Response Analysis and 8th Recognizing Textual Entailment Challenge* (Dzikovska et al., 2013). The corpus consists of two portions of student short-answers along with correct reference answers: The first portion, BEETLE, comprises student responses in the context of tutorial dialogues; the second, SciEntsBank comprises student answers to pre-selected science questions. For each pair of student and reference answers, the corpus is equipped with manual labels for 5-way (*correct*, *partially_correct_incomplete*, *contradictory*, *irrelevant* or *non_domain*), 3-way (*correct*, *contradictory* or *incorrect*) or 2-way (*correct* or *incorrect*) classification.

Recent work using data from SRA include Riordan et al. (2017), Sung et al. (2019) and Li et al. (2021). Direct comparison between these results are difficult, however: Riordan et al. (2017) worked on 5-way and 2-way classification on the full SRA dataset; Sung et al. (2019) addressed 3-way classification on the SciEntsBank portion only; Li et al. (2021) worked on the full dataset with all three label sets. Riordan et al. (2017) reported weighted F1-scores across all labels, while Li et al. (2021) used macro-average F1-scores and Sung et al. (2019) reported both.

As in the case of essay scoring, datasets with respect to non-English ASAS are scarce. However, efforts at creating publicly available resources exist. Examples include Mizumoto et al. (2019)’s dataset for Japanese and ASAP-DE (Horbach et al., 2018) and ASAP-ZH (Ding et al., 2020) for German and Chinese, respectively.

## Conclusion

This survey has provided an overview of supervised ML and DL approaches to student free-text evaluation in recent years. We considered feature-based models, neural and hybrid approaches to the task and reviewed recent studies in the field, providing detailed examples of model architectures, data and use cases.

Based on our research, we consider the following general insights as noteworthy:

- Fine-grained and comprehensive evaluation of student texts, especially longer essays, remains a challenging task. Several studies we reviewed use elaborate systems to evaluate a single aspect of essays, such as discourse structure (Šnajder et al., 2019; Song et al., 2020) and organisation (Song et al., 2020). This points to the difficulty of developing a holistic model that provides detailed evaluation from multiple relevant perspectives. This is also reflected in Ramesh and Sanampudi (2021)’s observation that essay scoring systems addressing all parameters including cohesion, coherence, prompt relevance etc. are rare.
- Aside from simply providing a score or assessment, works like (Hellman et al., 2020) and (Mizumoto et al., 2019) have put emphasis on explaining or justifying the model’s evaluation to the student. Not only is this interesting from the viewpoint of explainable AI; it is particularly relevant to tutoring tools that can encourage students to understand and learn from past errors.
- Compared to the earliest neural approaches (Alikaniotis et al., 2016; Taghipour & Ng, 2016), more recent works like those by Zhang and Litman (2018), Nadeem et al. (2019) and Yang and Zhong (2021) have shown attempts to incorporate wider contexts into neural representations of students’ sentences, whether from neighbouring sentences or additional textual material.
- Neural approaches, particularly those based on pre-training, are highly successful (Xue et al., 2021). Nonetheless, hand-crafted features remain relevant, especially when combined with neural features in hybrid systems (Kumar et al., 2020; Uto et al., 2020).
- Many challenges remain: For ASAS, adversarial student texts that represent possible cheating attempts continue to pose difficulties, also to the recent models, as shown by Ding et al. (2020). With respect to AEE, Jeon and Strube (2021) found that essay scoring systems can be overly influenced by the correlation between essay length and essay quality, while length is not necessarily an indicator of quality.

Further research is clearly needed in the field, especially for non-English data, for which work is scarce. Fine-grained and accurate evaluation of both short and essay-length free-texts by students are crucial to building intelligent educational applications and as such are likely to remain of great interest in the years to come.

## Footnotes

1 See, for instance, https://digital-strategy.ec.europa.eu/en/policies/digital-learning, https://www.worldbank.org/en/topic/edutech

2 The authors refer to their video demonstration at https://vimeo.com/238406360 to illustrate the user experience with the tool.

3 https://www.korbit.ai/

4 See Sherstinsky (2020) for a description of RNNs in general.

5 See Schuster and Paliwal (1997) for a detailed description of bi-directional RNNs.

6 See Goodfellow et al. (2016) for a discussion of CNNs in general.

7 The word *port* has multiple unrelated interpretations, among others: a) that of a synonym to *harbour* and b) that of the sweet Portuguese wine.

8 The field is more commonly know as *automatic essay scoring*. We use the term *evaluation* instead of *scoring* to emphasise that our survey is not limited to models that predict *scores*.

9 http://www.kaggle.com/c/asap-aes

10 See, for instance, Ke and Ng (2019) for a list of essay scoring datasets and Horbach and Zesch (2019) for ASAS datasets.

11 https://www.kaggle.com/c/asap-aes

12 https://www.kaggle.com/c/asap-sas

13 However, their work is on scoring specific essay traits instead of predicting the holistic score, see “Evaluation of Aspects of Student Essays”.

14 As described on Kaggle (https://www.kaggle.com/c/asap-aes/overview/evaluation), QWK scores typically range between 0 (random agreement between annotators) and 1 (complete agreement), although a negative score for agreement below chance is possible too. QWK factors in the *extent* to which two annotations for a specific sample disagree, which is clearly useful in the scoring context due to the ordered nature of scores. Thus, on a 4-point scoring scale, for instance, predicting a score-4 sample as having a score of 3 is certainly not as bad as predicting a score of 1 for that sample.

15 https://catalog.ldc.upenn.edu/LDC2014T06
